# S100A1 is a Potential Biomarker for Papillary Thyroid Carcinoma Diagnosis and Prognosis

**DOI:** 10.7150/jca.51855

**Published:** 2021-07-30

**Authors:** Ge Wang, Han-ning Li, Xiao-qing Cui, Tao Xu, Meng-lu Dong, Shu-yu Li, Xing-rui Li

**Affiliations:** 1 Department of Thyroid and Breast Surgery, Tongji Hospital, Tongji Medical College of Huazhong University of Science and Technology, Wuhan, Hubei 430030, People's Republic of China.; 2 Department of Obstetrics and Gynecology, Cancer Biology Research Center, Tongji Hospital, Tongji Medical College of Huazhong University of Science and Technology, Wuhan, Hubei 430030, People's Republic of China.

**Keywords:** Calcium binding proteins (CBP), Hippo, papillary thyroid carcinoma (PTC), S100A1, yes associated protein (YAP).

## Abstract

S100 calcium binding protein A1 (S100A1) is an important member of the S100 family and known to express in a variety of cancers. However, the biological functions of S100A1 in thyroid carcinoma have not been thoroughly studied. In this report, bioinformatics analyses and immunohistochemistry assays were applied to assess the expression profile of S100A1 as well as its relationship with the pathological features and prognosis of papillary thyroid carcinoma (PTC). Meanwhile, functions of S100A1 in PTC cells were analyzed with either *in vitro* or *in vivo* experiments. S100A1 was significantly up-regulated in PTC tissues compared with adjacent non-cancerous tissues. S100A1 protein expression was significantly associated with tumor size (*p*=0.0032) or lymph node metastasis (*p*=0.0331). More importantly, an elevated S100A1 expression was significantly correlated with a worse recurrence-free survival (RFS) (HR=2.26, *p*=0.042). Further, knockdown of S100A1 dramatically inhibited cell proliferation and migration as well as increased apoptosis of PTC cells. S100A1 knockdown inhibited tumor progression as seen in* in vivo* experiments. In terms of mechanism, down-regulation of S100A1 induced yes associated protein (YAP) phosphorylation in the cytoplasm and diminished Hippo/YAP pathway activation. Therefore, S100A1 may serve as a novel oncogene and a promising biomarker for PTC diagnosis and prognosis.

## Introduction

Diagnosis of thyroid neoplasm is growing fast worldwide 1. Majority of the new cases has been attributed to an increase in papillary thyroid carcinoma (PTC) 2, 3, which usually has a good prognosis. Although the etiology of PTC remains unclear, increased use of diagnostic imaging has been identified as one of the major driving forces 4, 5. The increased use of diagnostic imaging leads to an over-diagnosis of PTC, which may put patients at risk for over-treatment without conferring a survival benefit 6. In order to promote individualized treatments, new biomarkers are urgently needed to distinguish PTC patients who need from those who do not need aggressive treatments.

The S100 calcium binding protein family has more than 20 members. By functioning both as extracellular signaling factors and intracellular Ca^2+^ sensors 7, they are involved in a variety of cellular biological processes, including cell proliferation, transformation, invasion, and migration 8, 9. Multiple S100 protein family members, like S100A4 10-12, S100A8 13, S100A9 14, S100A12 15, S100A13 16, have been reported to be involved in the initiation and prognosis of thyroid neoplasms, which suggests a close and complex relationship between S100 proteins and PTC.

Many S100 protein family members including S100A2 17, S100A6 17, S100A8 13, S100A11 18, and S100A13 16 play crucial roles in thyroid cancer development and progression, thus can be potential biological markers or therapeutic targets of PTC. It was reported that S100A4 overexpression is a poor prognostic biomarker for thyroid cancer 12. S100A4 knockout significantly inhibits the survival and invasion of anaplastic thyroid cancer cells and potentiates the effect of vemurafenib on tumor regression 10, 19. Overexpression of S100A4 is also associated with papillary thyroid carcinoma invasion and metastasis. Therefore, S100A4may be a potential target for PTC therapy 11, 20. Further, S100A12 expression is strikingly increased in PTC and strongly associated with TNM stage, tumor size, and lymph node metastasis. Silencing S100A12 reduced the aggressive growth of tumor cells with either *in vitro* or *in vivo* experiments 15.

S100A1, also called S100 alpha, is the first discovered member of the S100 family. Previously studies showed that S100A1 could be detected in many tissues. For example, S100A1 is expressed in both heart and skeletal muscle. S100A1 silencing reduces contractility of cardiomyocytes. By contrast, enhanced cardiac S100A1 expression increases energy production and contractility 21, 22 and improves recovery of the heart from ischemia-reperfusion injuries 23, 24. S100A1 is also highly expressed in various cancer cells and participates in regulating important pathophysiological processes. For example, expression level of S100A1 is markedly increased in melanoma tissues compared with normal skin tissues 25. S100A1 is abnormally expressed and serves as an independent prognostic indicator for relapse-free survival in endometrioid subtypes of ovarian carcinoma 26, 27. However, the relationship between S100A1 and PTC is still unclear. In this study, S100A1 expression in human PTC was investigated to determine its diagnostic utility in thyroid cancers. Prognostic value of the biomarker in PTC patients was also evaluated. Meanwhile, its oncogene role was analyzed further with *in vitro* or *in vivo* experiments.

## Materials and Methods

### Patients and specimens

86 pairs of papillary thyroid carcinoma tissues and matched adjacent normal thyroid tissues were collected from patients who underwent surgical procedures at Tongji Hospital of Huazhong University of Science and Technology (Wuhan, China) between April and October 2019. Histopathological diagnoses of all tissue samples were made by three pathologists independently. No patients received any therapy before operation. Our study has been approved by the Research Ethics Committee of the Tongji Medical University, and all patients signed their informed consent forms.

### Immunohistochemistry

Tissue samples were fixed in paraformaldehyde and embedded in paraffin. After conventional sectioning, de-waxing, and hydrating steps, tissues received antigen repair using citric acid buffer. Endogenous peroxidase was blocked using 3% hydrogen peroxide for 30 minutes. The tissue sections were treated with S100A1 primary antibody (Novus, NBP2-29403, 1:400) overnight at 4℃ then incubated with horseradish peroxidase-conjugated goat anti-mouse IgG secondary antibody (servicebio, G1216-200T, 1:200) for one hour at room temperature, and then stained with 3,3′-diaminobenzidine (DAB) (servicebio, G1216-200T). Hematoxylin was used to stain nucleus. All immunohistochemical procedure was conducted under the same conditions. Scoring of IHC was performed using the method described by Zhang *et al* 28. Briefly, both percentage and staining intensity of tumor cells were scored separately. Score 0 (percentage of positive cells<5%); Score 1 (percentage of positive cells 5%-25%); Score 2 (percentage of positive cells 26%-50%); Score 3 (percentage of positive cells 51%-75%); Score 4 (percentage of positive cells>75%). Scale of 0-3 was used for grading staining intensity. Multiplication of the two scores was regarded as the final staining score. For statistical analyses, patients who had a negative or weak S100A1 expression were assigned to the low expression group (score≤4). By contrast, patients with a strong S100A1 expression were assigned to the high expression group (score>4).

### Cell culture

Human PTC cell lines K1, TPC1, BCPAP were purchased from the American Type Culture Collection (ATCC; Manassas, VA, USA). The normal thyroid follicular epithelium cell line (Nthy-ori3-1) was purchased from Chinese Academy of Sciences (Shanghai, China). Short tandem repeat (STR) and amelogenin of all these cell lines had been identified by a third party (Shanghai BioWing). K1, TPC1 and Nthy-ori3-1 were cultured in 10% (v/v) fetal bovine serum (FBS)-supplemented 1640 medium (Gibco, Grand Island, USA). BCPAP cells were cultured in 1640 medium with 1× Non-Essential Amino Acids Solution (NEAA; Life Technologies) and 10% FBS. Cells were cultured in the incubator at 37 degree Celsius (°C) containing 5% CO2. Culture medium was changed every other day, and cells were passaged when the density reached about 80% confluence.

### qRT-PCR

The total RNA was extracted from PTC cells by using Trizol reagent (Invitrogen), after the quality authentication of RNA concentration via 260/280nm absorbance, cDNA was synthesized using reverse transcription kit (Vazyme). The quantitative real-time PCR (qRT-PCR) was conducted by using the Bio-Rad CFX96 PCR system and the SYBR Green procedure. Primers were all determined through national center for biotechnology information (NCBI) and purchased from the Tsingke company, the human S100A1 primers were 5'-GACCCTCATCAACGTGTTCCA-3' (forward) and 5'-CCACAAGCACCACATACTCCT-3' (reverse), the Glyceraldehyde 3-phosphate dehydrogenase (GAPDH) primers were 5'-GGAGCGAGATCCCTCCAAAAT-3' (forward) and 5'- GGCTGTTGTCATACTTCTCATGG-3' (reverse). Relative mRNA expression levels were calculated by using the 2^-ΔΔCt^ method normalized to GAPDH (internal control).

### Western blot assay

PTC tissues as well as paired normal thyroid tissues were cut and then pulverized under liquid nitrogen. Cells were lysed with cold RIPA buffer supplemented with protease inhibitor cocktail (Servicebio), followed by ultra-sonication at low frequency and centrifugation to collect the supernatant lysate. The protein concentration was determined by Coomassie Brilliant Blue method. Following the manufacturer's protocol, the nuclei and cytoplasm proteins in PTC cells were separated using the cytoplasm and nuclear protein extraction kit (KeyGen Biotech). Twenty micrograms of proteins were separated on 10% SDS-PAGE gels and then transferred onto polyvinylidene fluoride (PVDF) membranes. Primary antibodies were diluted in 1% bovine serum albumin (BSA) and added to the PVDF membranes, which were incubated at 4℃ overnight with the following antibodies: S100A1 (Novus, NBP2-29403, 1:200), YAP (Immunoway, YT4924, 1:500), P-YAP (Immunoway, YT0708, 1:500), GAPDH (AntGene, ANT011, 1:5000), Histone H3 (Abcom, ab1791, 1:500). The PVDF membranes were then incubated with horseradish peroxidase (HRP) goat anti-rabbit (AntGene, ANT020, 1:2000) or HRP goat anti-mouse (AntGene, ANT19, 1:2000) secondary antibody. The proteins were detected using the WesternBright™ ECL kit (advansta).

### Cell transfection

S100A1-RNAi siRNA were purchased from Guangzhou ribobio Company (Guangzhou, China). The target sequences for S100A1-Si1, S100A1-Si2 and S100A1-Si3 were GGAGCTAGACGAGAATGGA, CCAGGAGTATGTGGTGCTT, ACAGTGGCCTGTAACAATT respectively. Thyroid cancer cells were transfected according to the manufacturer's protocol. Silencing efficiency was evaluated by qRT-PCR and Western blot (48 and 72 hours after transfection respectively). The S100A1 lentivirus and negative control lentivirus were purchased from Genechem company (Shanghai, China). The S100A1-shRNA1 target sequence was GGAGCTAGACGAGAATGGA, the S100A1-shRNA2 target sequence was ACAGTGGCCTGTAACAATT, and the negative control shRNA was TTCTCCGAACGTGTCACGT. These DNA oligonucleotides were inserted into hU6-MCS-Ubiquitin-EGFP-IRES-puro lentiviral vector. Transfection efficiency of lentivirus was assessed with qRT-PCR and Western blot assays.

### Cell Counting Kit-8 and Colony formation assays

Cell growth rate was assessed by Cell Counting Kit-8 (CCK-8) assays. 3,000 cells were seeded per well in a 96-well plate at 0 hour, and the absorbance was measured every 24 hours for 4 days. Before testing started, the medium was changed to 1640 medium containing 10% CCK-8 (100ul/well, Dojindo, Japan). After incubated for one hour at 37°C, the optical density (OD) value was measured using the microplate reader at the wavelength of 450nm. Colony formation assays were conducted to detect the ability of a single cell to grow into a colony. PTC cells were seeded in a six-well plate at the same density (2,000 cells/well). After cultured for 14 days, the clones were fixed by 4% paraformaldehyde and stained with 0.1% crystal violet. Visible PTC cell clones were counted using the ImageJ software.

### Cell migration assays

The migration capacity of cells was evaluated by using the 24-well transwell chamber with 8μm pore sizes (Corning, USA). Cells (2×10^4^ cells/well) were seeded in the upper chambers in serum-free medium, and the lower chambers were filled with 1640 medium containing 15% FBS as a chemo-attractant. After being cultured in a 37°C incubator for 12 hours, PTC cells in the upper chambers were carefully cleaned, while invaded PTC cells at the lower membrane surface were fixed with 4% paraformaldehyde and stained with crystal violet. Finally, those cells were sealed with neutral resin and observed under a microscope. Three visual fields in each sample were randomly selected to count the invaded cells.

### Flow cytometric analysis

Apoptosis was measured by Annexin V/PI staining using the Apoptosis Detection Kit (BD Biosciences). PTC cells were stained with Annexin V-FITC and PI by following the instructions, and then examined by means of the FACScan flow cytometer. The percentage of apoptotic cells was calculated as the sum of the early and late apoptosis rates. For cell cycle experiments, cells were washed with cold PBS and fixed in 85% ethanol at -20℃ overnight. RNase (8 μg/ml, AntGene) and propidium iodide (50 μg/ml, Sigma Aldrich) were used to treat cells at 37 °C for 30 minutes. FACScan flow cytometer was used for analyzing the cell cycle distribution.

### Nude mouse xenograft model

All mouse care and operations were carried out in accordance with the Tongji Medical University Institutional Animal Care and Use Committee regulations. Five-week-old female BALB/c nude mice were purchased from Charles River Laboratories (Beijing China). 100 ul sterile PBS containing 1×10^7^ cells mixed with 100 ul Matrigel Matrix (356234, Corning), which was carefully injected subcutaneously into the right armpit of each nude mouse. Tumor dimensions were monitored every three days to measure tumor sizes utilizing the following formula: volume=1/2(length×width^2^). 24 days later, all the nude mice were sacrificed. Those tumor tissues were removed and subjected to H&E staining and IHC studies.

### TCGA data acquisition and analysis

The raw RNA sequencing data and corresponding clinical information of thyroid carcinoma patients was obtained from The Cancer Genome Atlas (TCGA) database. S100A1 mRNA expression data were extracted as well. For all subsequent analyses, original data were log2 transformed for normalization, and then the difference between cancer and thyroid was estimated. We also analyzed multiple tissue subtypes of PTC to analyze whether there was a relationship between the expression of S100A1 and PTC subtypes. In addition, differential CpG islands methylation expression in the promoter region of the S100A1 gene was analyzed by querying the UALCAN portal. Relative methylated levels of thyroid cancer and normal thyroid tissues were presented in units of beta values, ranging from 0 (no methylation) to 1 (full methylation).

### Survival analysis of S100A1

The prognostic values of S100A1 in patients with thyroid cancer were evaluated by Kaplan-Meier plotter (KM-plotter) online database. According to the median expression value of S100A1, all thyroid cancer patients were divided into high-expression and low-expression groups, and the recurrence-free survival (RFS) rate was calculated. The hazard ratio (HR) and 95% confidence interval (CI) were calculated and reported, and the curves were statistically compared using the log-rank test. We followed up 86 PTC patients until November 2020 via telephone calls. Patients were divided into S100A1 high and low expression groups according to median S100A1 expression level. Kaplan-Meier survival analysis was performed and expressed as survival rate in percentage.

### Statistical analysis

Statistical analyses were performed using the SPSS (version 20; SPSS Inc. USA), and graphs were made with the GraphPad Prism (version 8.00; GraphPad Software Inc.). Receiver operating characteristic (ROC) curves, area under the curve (AUC), the sensitivity and specificity were also performed using GraphPad Prism 8. All experiments were performed independently for at least three times. Means of two matched/unmatched groups were compared using the paired/unpaired T tests. One-way ANOVA or two-way ANOVA were utilized for comparing the means of three or more comparison groups. P< 0.05 was used to indicate a statistically significant difference.

## Results

### S100A1 is up-regulated in PTC tissues and correlated with pathological features

**Figure 1:**
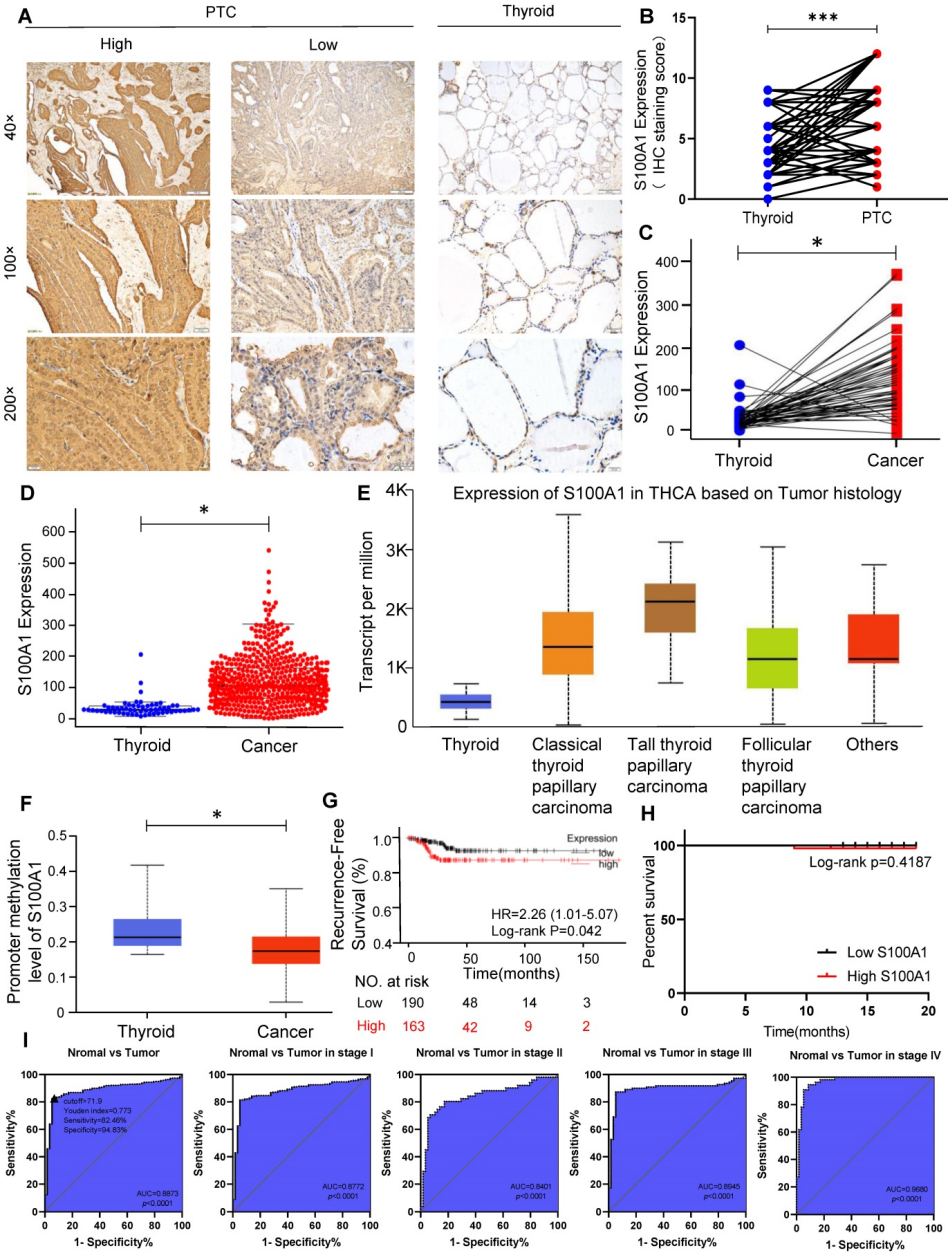
High expression of S100A1 in PTC might be a new biomarker. (A): Immunohistochemistry (IHC) staining of S100A1 was shown in human PTC and normal thyroid tissues. (B): IHC staining score of S100A1 was compared in human PTC and normal thyroid tissues (***P<0.001). (C-D): S100A1 expression was compared in paired samples and population samples in TCGA database, respectively. (*P<0.05). (E): Comparison of the expression of S100A1 in normal thyroid and various PTC subtypes in TCGA. (F): Methylation levels of S100A1 gene promoter in thyroid cancer were lower than normal thyroid based on TCGA data, which were analyzed using UALCAN web. (*P<0.05). (G): Kaplan-Meier analysis showed that a higher expression of S100A1 was associated with a worse recurrence-free survival. (H): Survival analysis was shown based on our follow-up data of 86 patients. (I): ROC curves were shown for discrimination of normal tissues and PTC tissues. An optimal cut-point derived from the maximum of Youden index was displayed as a triangle (Youden index: 0.773; Sensitivity: 82.46%, Specificity: 94.83%). ROC curves for diagnosis in stage I, stage II, stage III, and stage IV patients. PTC, papillary thyroid carcinoma; TCGA, The Cancer Genome Atlas; ROC, receiver operating characteristic; AUC, area under the curve.

Correlation between S100A1 protein and pathological features were investigated using tissue samples from PTC patients. IHC showed S100A1 staining was positive both in the nucleus and cytoplasm of PTC cells, while the S100A1 staining was negative or weak in the normal adjacent tissues **(Fig. 1A)**. S100A1 protein showed a higher expression in PTC tissues than in normal thyroid tissues** (Fig. 1B)**, which is in agreement with the bioinformatics analysis results **(Fig. 1C, 1D)**. No any other histological subtype of PTC was found in the specimens we collected. Thus, only classic PTC was studied in our research. We analyzed the expression of S100A1 in normal thyroid tissue and different histological subtype of PTC (classical PTC, tall cell PTC, follicular PTC) in TCGA database. As shown in **Figure 1E**, compared with normal thyroid tissue, the expression of S100A1 in various PTC subtypes was higher and the highest in the tall cell PTC. Methylation level of S100A1 gene promoter in thyroid cancer was lower than in normal thyroid tissue, which suggested difference in gene expression might due to different methylation levels **(Fig. 1F)**. Kaplan-Meier survival curve analysis showed that thyroid cancer patients expressing higher S100A1 had a less favorable RFS** (Fig. 1G)**. The telephone follow-up data for all 86 patients were as following: 10 patients could not be contacted, 75 patients were alive and recovered well after the surgery, and one patient died of heart disease. No evidence of local recurrence or distant metastasis was observed in these patients. These follow-up data were expected, probably due to the slow progression and indolent nature of the disease. We performed a survival analysis for DFS based on these data, and found no statistical difference between the S100A1 high and low group** (Fig. 1H)**. This is inconsistent with the results of the previous TCGA data analysis **(Fig. 1G)**, which may be due to the small sample size and a shorter follow-up. Associations between S100A1 protein expression levels and pathological features were summarized in **Table 1**. High expression of the S100A1 was associated with tumor size (*p*=0.0032), lymph node metastasis (*p*=0.0331), but not with gender, age, extrathyroidal extension or bilateral PTC. All these results indicated that S100A1 protein was up-regulated in PTC compared with matched normal thyroid tissues. Patients with high S100A1 expression were more likely to have more aggressive pathological features and a worse prognosis.

### S100A1 as a molecular biomarker for PTC diagnosis

ROC curves revealed that S100A1 exhibited an excellent diagnostic efficiency for PTC (AUC=0.8873, p<0.0001; **Fig. 1I**). The optimal cut-point by Youden index for the predicted threshold of S100A1 was an expression value of 0.719 for PTC (Youden index: 0.773; Sensitivity: 82.46%, Specificity: 94.83%). Moreover, the diagnostic efficiency of S100A1 had high quality among all clinical stages (stage I: AUC=0.8772, stage II: AUC=0.8401, stage III: AUC=0.8945, stage IV: AUC=0.9680; *p*<0.0001 for all), especially in stage IV, suggesting S100A1 as a fine molecular biomarker for PTC diagnosis.

### SiRNA transfection downregulates S100A1 protein in PTC cell lines

**Figure 2:**
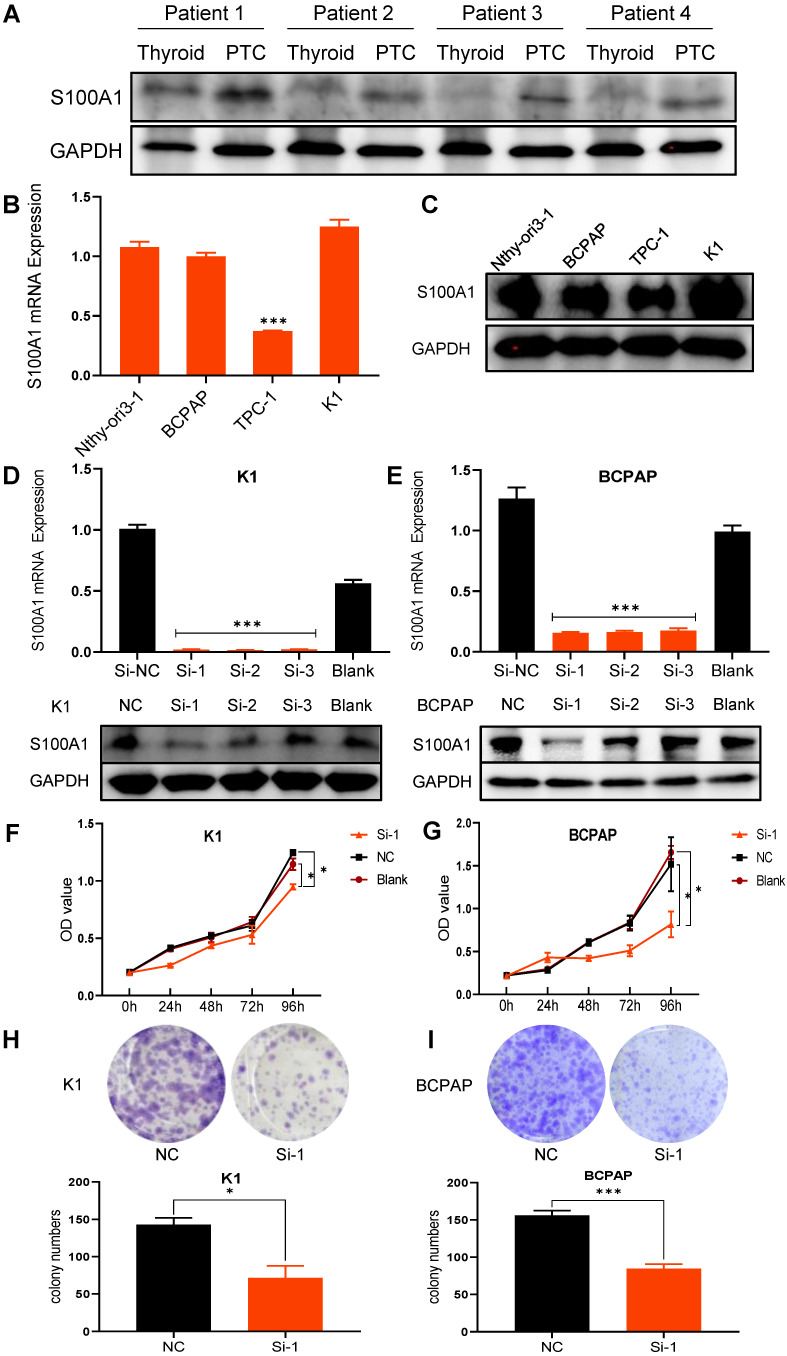
Knockdown of S100A1 inhibited cell proliferation. (A) Differential expression of S100A1 protein was compared between human PTC tissues and paired thyroid tissues. GAPDH: Glyceraldehyde 3-phosphate dehydrogenase. (B): The S100A1 mRNA expression was shown in four cell lines (Nthy-ori-3-1, BCPAP, TPC-1, and K1) (***P<0.001). (C): The protein expression of S100A1 in four cell lines was identified by Western blot assays. GAPDH: Glyceraldehyde 3-phosphate dehydrogenase. (D-E): The mRNA and protein expression of S100A1 were compared among three Si, the NC and the blank groups. Si-1 groups showed the lowest S100A1 protein expression in either K1 or BCPAP cell lines (***P<0.001). (F-G): Compared with the NC or blank groups, Si-1 groups showed the lowest growth rate both in either K1 or BCPAP cell lines (*P<0.05). OD value at 450 nm was measured by CCK-8 assay. (H-I): Compared to the NC group, the number of cell clones significantly decreased in Si-1 groups (*P<0.05, ***P<0.001). NC: negative control; OD: optical density.

In order to further explore the expression of S100A1 in thyroid and papillary thyroid carcinoma in humans, we collected 4 PTC tissues and paired adjacent normal thyroid tissues, and compared the expression of S100A1 protein by Western blot assays **(Fig. 2A)**. Consistent with the results of our previous immunohistochemical experiments, the expression of S100A1 in PTC was significantly higher than in normal thyroid. Basal expression of S100A1 in the normal thyroid follicular epithelium cell line (Nthy-ori3-1) and three PTC cell lines (BCPAP, TPC-1, K1) were examined by qRT-PCR and Western blot assays. TPC-1 cell line showed the lowest level, while BCPAP, K1 and Nthy-ori3-1 showed high levels of S100A1 expression **(Fig. 2B, 2C)**. Expression of S100A1 in Nthy-ori3-1 cell line was abnormally increased, which may be due to cell line dedifferentiation 29, 30. To validate the functions of S100A1 in PTC cells, we selected K1 and BCPAP, the two PTC cell lines with high S100A1 expression, for carrying out further experiments. S100A1 siRNA knockdown experiments were conducted to test its functions. Transfection efficiency was assessed via qRT-PCR and Western blot assays **(Fig. 2D, 2E)**. S100A1 expression was greatly decreased in siRNA groups compared to either blank or negative control groups (especially for the Si-1 groups), which indicated that S100A1 expressions were successfully knocked down.

### S100A1 regulates the tumorigenicity of PTC cells *in vitro*

**Figure 3:**
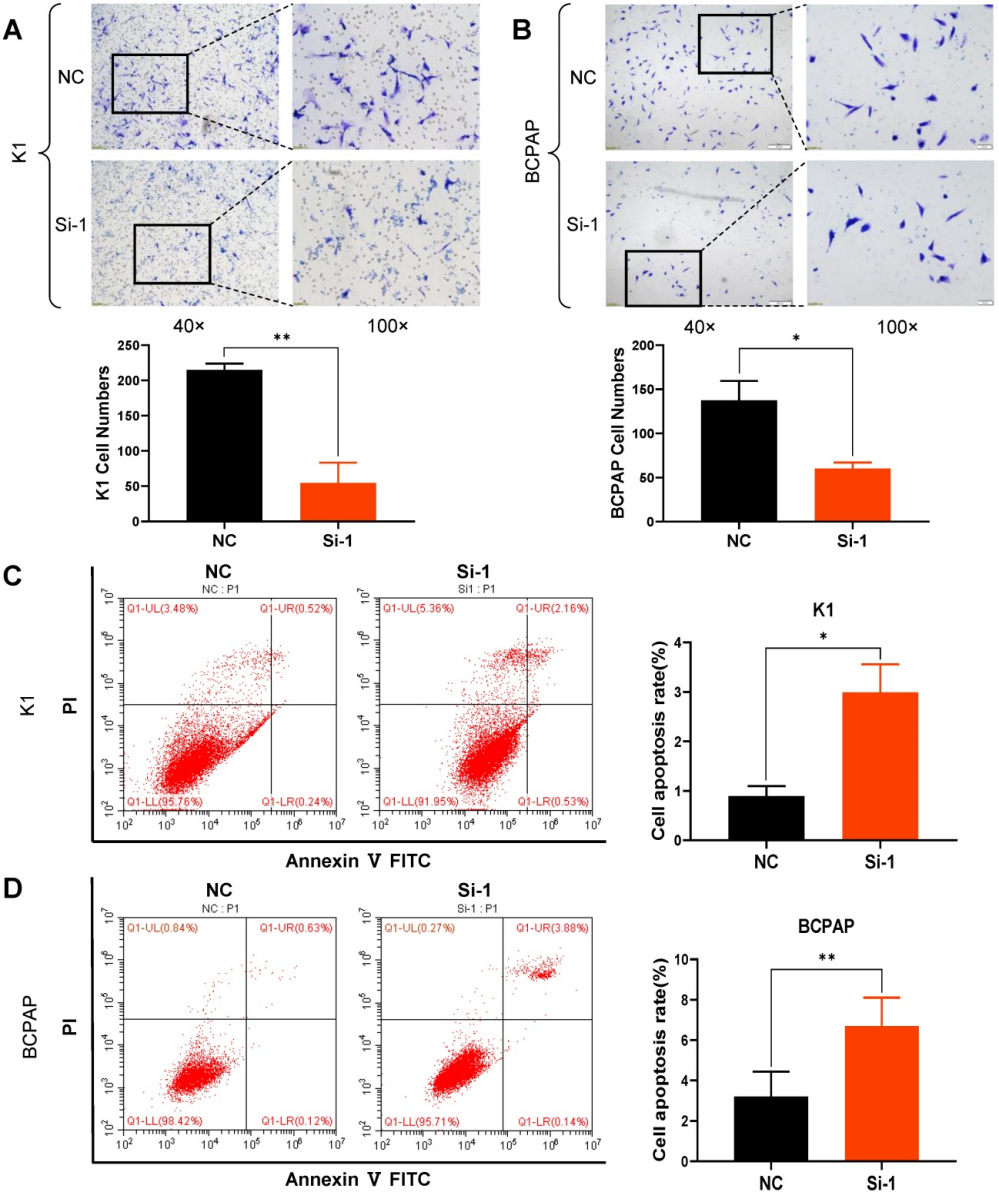
Knockdown of S100A1 inhibited cell migration and enhanced apoptosis. (A-B): Numbers of migrating cells in the Si-1 groups were less than in the NC group, and the cell numbers were analyzed for either K1 or BCPAP cell lines (*P<0.05, **P<0.01). (C-D): Apoptosis percentage in the Si-1 group was higher than the NC group, and the cell apoptosis rate was analyzed for either K1 or BCPAP cell lines (**P<0.01). NC: negative control.

CCK8 and colony formation assays were carried out in order to ascertain whether S100A1 affects cell proliferation. Cell growth curves indicated that down regulation of S100A1 significantly inhibited the proliferation of K1 and BCPAP cells when compared to control groups **(Fig. 2F and 2G)**. Consistent with the CCK8 results, colony formation assays revealed that colonogenic ability was also suppressed when S100A1 was knocked down **(Fig. 2H and 2I)**. Transwell assays were performed to see whether S100A1 also regulated cell migration. As shown in** Fig. 3A and 3B**, the number of cells that migrated into the lower surface in the Si-1 groups were less than the counterpart measure in the negative control (NC) groups for either K1 or BCPAP cell line. Taken together, these results indicated that proliferation and migration of PTC cells were inhibited by the S100A1 knockdown.

### S100A1 knockdown induces apoptosis of PTC cells

Many factors including cell senescence and apoptosis could influence cell proliferation. So S100A1 was studied for its possible effects on cell cycle and apoptosis. The percentages of apoptotic cells were significantly increased in Si-1 groups compared to the NC groups **(Fig. 3C and 3D)**. However, the proportion of cells in various phases of the cell cycle was not significantly different between the experimental groups and control groups (data not shown).

### S100A1 regulates the tumorigenicity of PTC cells

**Figure 4:**
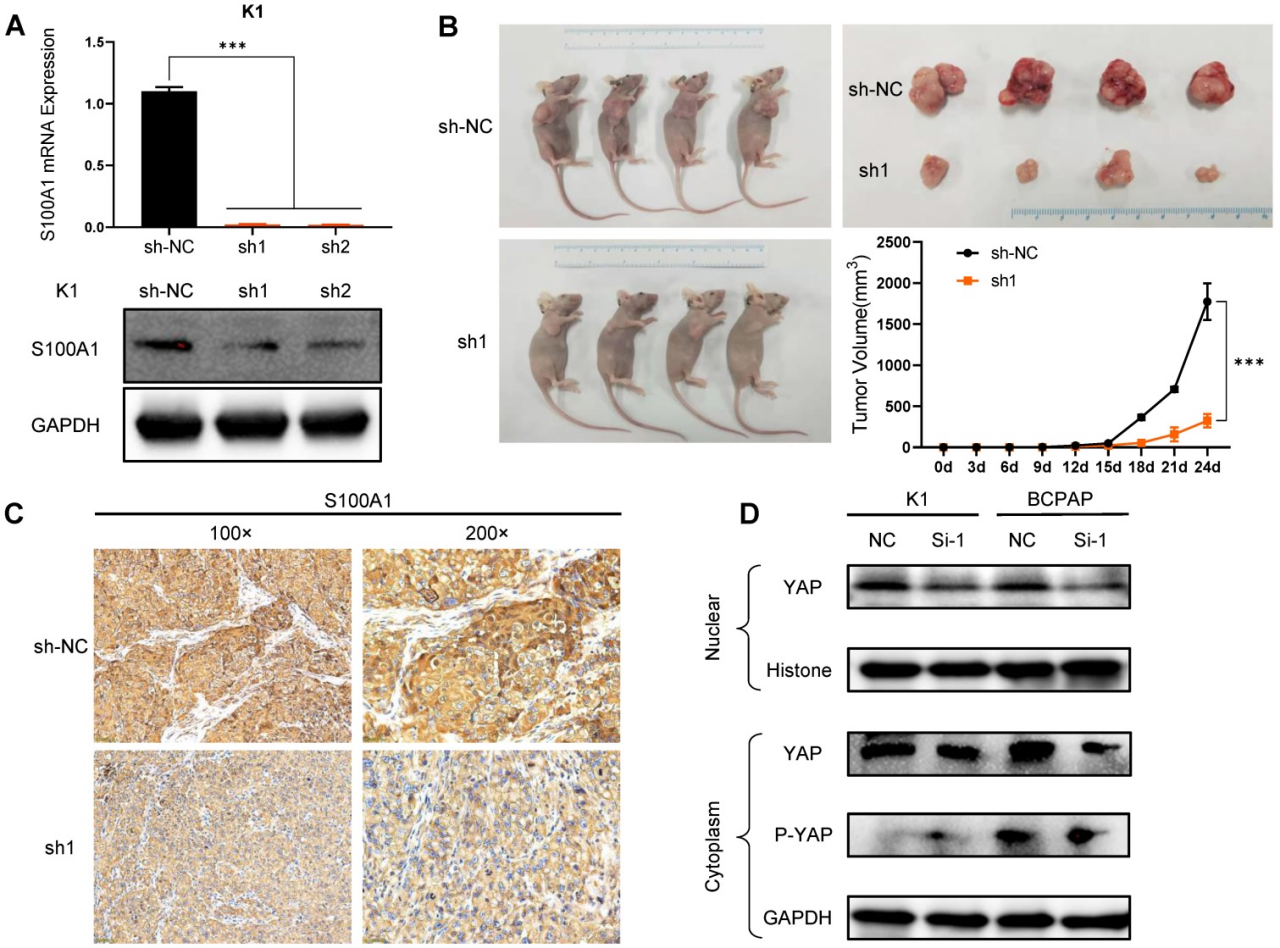
S100A1 regulates the tumorigenicity of PTC cells *in vivo* and the underlying molecular mechanism. (A): Constructed S100A1 was stably silenced in K1 cells via lentivirus (***P<0.001). (B): Mouse xenograft models and tumor tissues were measured 24 days after subcutaneous injection with K1-shNC cells or K1-sh1 cells. Tumor volumes were examined every three days for 28 days (*P<0.05). (C): IHC staining of S100A1 protein was shown in nude mice transplanted tumors. (D): Western blot assays of the nuclear and cytoplasmic YAP/P-YAP protein were shown. PTC: papillary thyroid carcinoma; IHC: immunohistochemistry; YAP: yes associated protein; P-YAP: phosphorylated YAP.

To further delineate the role of S100A1 in PTC, we performed *in vivo* experiments with nude mouse xenograft models using K1 cells. First, K1 cells with stably silenced S100A1 were constructed via lentivirus. qRT-PCR and Western blot assays were used to ensure the transfection efficiency after S100A1 specific shRNA transfection** (Fig. 4A)**. Compared with the NC group (sh-NC), sh1 and sh2 groups both had relatively lower mRNA and protein expression, and sh1 group had the lowest expression. Then, K1-sh1 and K1-shNC cells were chosen for performing animal experiments. The volume of tumors in sh1 group was measured 24 days after subcutaneous injection, which were significantly smaller than the counterpart measure in the sh-NC group **(Fig. 4B)**. Consistent with previous results, IHC data also showed the S100A1 expression in the sh1 group was lower than the sh-NC group **(Fig. 4C)**.

### Downregulation of S100A1 suppresses PTC cell growth through Hippo/YAP signaling

**Figure 5:**
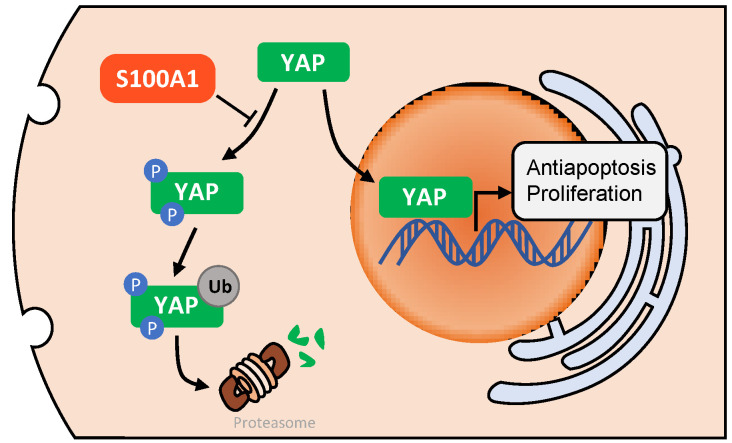
A diagram of the potential underlying regulatory mechanism of S100A1. After phosphorylation and ubiquitination, YAP is degraded by proteasomes in cell cytoplasm. S100A1 could suppress this process, resulting in YAP protein accumulation in cytoplasm and an increase in YAP entering the nucleus, and ultimately induces the transcription of antiapoptotic and proliferative genes. YAP: yes associated protein.

Many studies 31-33 have illustrated that Hippo/YAP pathway played an important role in tissue homoeostasis. Therefore, we investigated whether there was a relationship between S100A1 and Hippo/YAP signaling pathway. Through transient transfection in K1 and BCPAP cell lines, we found that the down regulation of S100A1 was always accompanied by a decrease of nuclear and cytoplasmic YAP protein. By contrast, knockdown of S100A1 increased the phosphorylated YAP in cytoplasm **(Fig. 4D)**. To sum up, our data suggested S100A1 might regulate the Hippo signaling pathway to promote PTC cells proliferation and suppress apoptosis **(Fig. 5)**.

## Discussion

The increased incidence of thyroid cancer is mainly attributed to detection of small PTC, which has a generally good prognosis 34. Of interest, with the similar histopathological features, PTC exhibited varied clinical features and prognoses. Still, about 10% of PTC patients develop metastasis and lead to fatal outcomes 35. Therefore, novel biomarkers for PTC diagnosis and prognostic assessment are urgently needed.

S100A1 is a member of the S100 calcium binding proteins family. Similar to most S100 family proteins, conformational changes take place in S100A1 upon binding calcium and interacting with many target proteins 8. Through specific interactions, S100A1 is involved in many pathophysiological processes such as synapse release 36, cytoskeletal proteins dynamics 32, and regulation of transcription factors 37. S100A1 was also reported to be involved in the occurrence and progression of tumors. For example, S100A1 is a novel biomarker in differentiating prostatic adenocarcinoma from nephrogenic adenoma 38. S100A1 was also suggested to be an immunohistochemical biomarker to distinguish chromophobe renal cell carcinoma from benign renal oncocytoma 39, 40. Li *et al.* found that S100A1 was expressed significantly higher in melanoma samples than in normal skin samples, which is also associated with clinical pathology such as lymphatic and distant metastasis 25.

**Figure 6:**
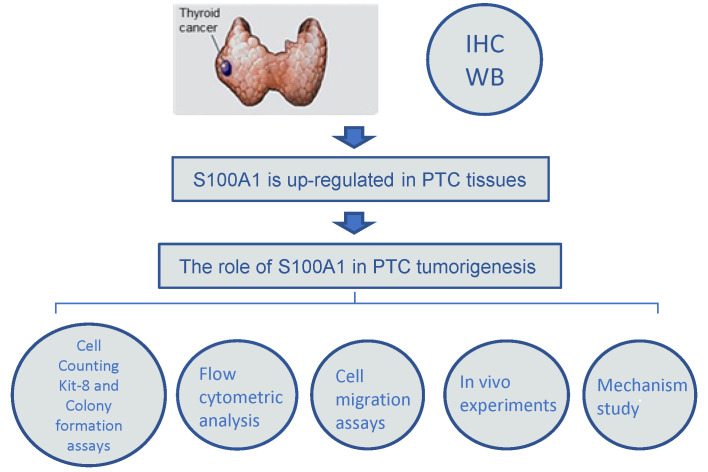
A simplified flowchart illustrating the various experimental steps of the study. Immunohistochemistry and Western blot assay findings showed that S100A1 protein was up-regulated in PTC samples. The role of S100A1 in PTC tumorigenesis was investigated by Cell Counting Kit-8 and colony formation assays, flow cytometric analysis, cell migration assays, and *in vivo* experiments.

A simplified flowchart illustrating the various experimental steps of the study was shown in Fig. 6. In brief, we first analyzed the differential expression of S100A1 in human PTC and normal thyroid tissue. IHC and Western blot assay findings showed that S100A1 protein was remarkably up-regulated in PTC samples and associated with corresponding pathological changes. Additionally, a series of cellular functional experiments were performed to explore the role of S100A1 in PTC, such as CCK-8 assays, colony formation assays, cell migration assays and flow cytometric analysis. Animal experiments also demonstrated that knockdown of S100A1 inhibited PTC development. Finally, the molecular mechanisms behind these differences were explored.

The discrepancy on S100A1 protein expression may be due to different tissue types. Our IHC and Western blot assay data showed that S100A1 protein had markedly increased expression in PTC tissues than normal thyroid tissues, which suggests S100A1 may act as an oncogene in PTC. In addition, the ROC curves suggested that a high S100A1 expression was able to distinguish PTC from healthy subjects among all clinical stages, for serving as a robust diagnostic biomarker for PTC. Survival curve analysis based on TCGA dataset showed that the long-term prognosis was quite different between the S100A1 high and low groups, which suggested S100A1 can be used as a prognostic marker for cancer. However, our own survival data did not match TCGA dataset analysis, which may be due to limitations of a small sample size and a shorter follow-up. We will continue to monitor the disease progression of each patient in the studied cohort.

The functions of S100A1 in PTC cell lines were further shown with CCK-8 and colony formation assays, which indicated that S100A1 knockdown inhibited PTC cells proliferation *in vitro*. Moreover, the transwell assays demonstrated that PTC cells migration was inhibited by S100A1 silencing. Knocking down S100A1 inhibited the progression of PTC cells. Abnormal cell cycle and cell apoptosis are causes of tumorigenesis and cancer progression. Experiments were designed to investigate the reasons for a slow proliferation, in which S100A1 knockdown induced apoptosis but did not have a significant effect on cell cycle.

To confirm our findings with *in vivo* experiments, we analyzed the impact of S100A1 silencing in xenograft tumor nude mouse models. Tumors with S100A1 knockdown were comparatively small, which indicated that cell proliferation was greatly inhibited by S100A1 shRNA treatment compared to the negative control. Knockdown of S100A1 could suppress PTC cells *in vivo*, which suggests S100A1 may act as a novel oncogene in the development and progression of PTC.

To further explore the potential signaling pathway underlying PTC progression, we investigated the association between S100A1 and Hippo signal pathway through measuring expressions of phosphorylated YAP and total YAP. The Hippo/YAP signaling pathway plays a major role in cell proliferation and tissue homoeostasis. The pathway is a protein kinase cascade. With receiving an activating signal, the mammalian Ste20-like kinases 1/2 (MST1/2) is phosphorylated, which activates large tumor suppressor (LATS1/2), and then inhibits Yes-associated protein (YAP) phosphorylation. It was reported S100A1 inactivated the Hippo signaling pathway in hepatocellular carcinoma 41. Specifically, down-regulation of S100A1 reduced total and nuclear YAP protein but induced YAP phosphorylation in the cytoplasm. Down-regulation of S100A1 upregulated the phosphorylation of LATS1 but did not change the expressions of MST1/2 or p‑MST1/2 41. Additionally, S100A1 protein could promote chondrogenesis and chondrocyte phenotype maintenance by inhibiting Hippo/YAP pathway via LATS kinases and IQGAP1 (IQ motif containing GTPase activating protein 1) 32, 42. Our findings are consistent with previous reports. Huang et al. found that YAP expression was significantly correlated with the TNM stage, lymph node metastasis, and inferior recurrence-free survival in patients with PTC 43. YAP also plays an important role in PTC cellular proliferation and anti-autophagy 44, 45.

## Conclusion

In summary, our findings strongly support that S100A1 promotes papillary thyroid carcinoma cells proliferation and migration through regulating the Hippo/YAP signaling pathway, and functions as a novel biomarker for diagnosis and prognosis of papillary thyroid carcinoma. Inhibitor of S100A1 could also provide a new approach for treating papillary thyroid cancer.

## Figures and Tables

**Table 1:** The relationship between S100A1 expression and pathologic features of 86 papillary thyroid carcinoma patients

	N=86	S100A1		X^2^	P value
		High=52	Low=34		
**Gender**					
Male	18	12	6	0.3662	0.5451
Female	68	40	28		
**Age(year)**					
<45	35	22	13	0.1413	0.7070
≥45	51	30	21		
**Extrathyroidal extension**					
Yes	9	4	5	1.079	0.2989
No	77	48	29		
**Tumor size**					
<1cm	49	23	26	8.717	0.0032*
≥1cm	37	29	8		
**Bilateral PTC**					
Yes	15	11	4	1.259	0.2619
No	71	41	30		
**Lymph node metastasis**					
Yes	50	35	15	4.543	0.0331*
No	36	17	19		

Statistical significance was tested using Chi-square analysis (* P<0.05).

## Abbreviations

ATCC: American Type Culture Collection
IHC: immunohistochemistry
IQGAP1: IQ motif containing GTPase activating protein 1
LATS1/2: large tumor suppressor 1/2
MST1/2: mammalian Ste20-like kinases 1/2
PTC: papillary thyroid carcinoma
P-YAP: phosphorylated Yes-associated protein
RFS: recurrence-free survival
STR: Short tandem repeat
TCGA: The Cancer Genome Atlas
YAP: Yes-associated protein
